# Structural Changes in Polymeric Gel Scaffolds Around the Overlap Concentration

**DOI:** 10.3389/fchem.2019.00317

**Published:** 2019-05-08

**Authors:** Han Zhang, Matthew D. Wehrman, Kelly M. Schultz

**Affiliations:** Department of Chemical and Biomolecular Engineering, Lehigh University, Bethlehem, PA, United States

**Keywords:** multiple particle tracking microrheology, hydrogel scaffolds, poly(ethylene glycol), photopolymerization, time-cure superposition

## Abstract

Cross-linked polymeric gels are an important class of materials with applications that broadly range from synthetic wound healing scaffolds to materials used in enhanced oil recovery. To effectively design these materials for each unique applications a deeper understanding of the structure and rheological properties as a function of polymeric interactions is required. Increasing the concentration of polymer in each scaffold increases physical interactions between the molecules that can be reflected in the material structure. To characterize the structure and material properties, we use multiple particle tracking microrheology (MPT) to measure scaffolds during gelation. In MPT, fluorescently labeled probe particles are embedded in the material and the Brownian motion of these particles is captured using video microscopy. Particle motion is related to rheological properties using the Generalized Stokes-Einstein Relation. In this work, we characterize gelation of a photopolymerized scaffold composed of a poly(ethylene glycol) (PEG)-acrylate backbone and a PEG-dithiol cross-linker. Scaffolds with backbone concentrations below and above the overlap concentration, concentration where polymer pervaded volume begins to overlap, are characterized. Using time-cure superposition (TCS) we determine the critical relaxation exponent, *n*, of each scaffold. The critical relaxation exponent is a quantitative measure of the scaffold structure and is similar to a complex modulus, G^*^, which is a measure of energy storage and dissipation. Our results show that below the overlap concentration the scaffold is a tightly cross-linked network, *n*_*avg*_ = 0.40 ± 0.03, which stores energy but can also dissipate energy. As polymeric interactions increase, we measure a step change in the critical relaxation exponent above the overlap concentration to *n*_*avg*_ = 0.20 ± 0.03. After the overlap concentration the scaffold has transitioned to a more tightly cross-linked network that primarily stores energy. Additionally, continuing to increase concentration results in no change in the scaffold structure. Therefore, we determined that the properties of this scaffold can be tuned above and below the overlap concentration by changing the polymer concentration but the structure will remain the same in each concentration regime. This is advantageous for a wide range of applications that require scaffolds with varying stiffness and the same scaffold architecture.

## Introduction

Synthetic gels are designed with unprecedented complexity from the bulk material properties down to the scaffold microstructure (Stauffer et al., 1982; Moradi-Araghi et al., 1988; Scanlan and Winter, 1991; Lutolf et al., 2003; Engler et al., 2006; Serra et al., 2006; Zolfaghari et al., 2006; Yamaguchi et al., 2007; He et al., 2009; Schultz et al., 2009a; Schwartz et al., 2010; Tse and Engler, 2010; Zustiak and Leach, 2010; Wylie et al., 2011; Tirrell, 2012; Jung et al., 2013; Tongwa et al., 2013; Purcell et al., 2014; Wang and Heilshorn, 2015; Escobar et al., 2017). This complexity grows out of the vast array of applications and high demand on gel versatility. Gels are part of everyday life from commonly used personal, fabric and home care products to exotic biomaterials designed to mimic the extracellular matrix (ECM) (Winter and Chambon, 1986; Scanlan and Winter, 1991; West and Hubbell, 1999; Raeber et al., 2005, 2007; Yamaguchi et al., 2007; Benton et al., 2009; Fairbanks et al., 2009b; Zustiak and Leach, 2010; Wylie et al., 2011; Wehrman et al., 2018). Cross-linked gels have also played a significant role in enhanced oil recovery (Moradi-Araghi et al., 1988; Zolfaghari et al., 2006; He et al., 2009; Jung et al., 2013; Tongwa et al., 2013). These materials are used to decrease permeability in high permeability zones near naturally fractured carbonates that require water shutoff but cannot be permanently plugged. Rheological measurement is a critical means for characterizing and validating gelation strategies and gaining insight into their structure and properties. Quantitatively identifying dynamic scaffold structure and properties and the relation to material function is crucial in advancing the design of these materials. These history-dependent systems are characterized during gelation to establish a quantitative framework to understand how polymeric interactions, i.e. overlap and entanglement, within macromer solutions change the gelation reaction and influence final material properties in a chain-growth system. We characterize the scaffold structure and rheological properties of a well-defined photopolymerized hydrogel scaffold with increasing polymeric interactions due to an increase in the concentration of the polymer backbone. This work can be leveraged to design gels with highly-engineered microstructures and properties that can be tailored throughout the phase transition.

In this work, polymeric interactions are varied to determine the change in the gelation reaction and final material properties. We are using a neutral polymer, poly(ethylene glycol) (PEG). Due to this, polymeric interactions are defined as only physical interactions between the macromolecules in solution. Polymeric solutions have three regimes in the concentration-viscosity curve: dilute, semi-dilute and entangled (Graessley, 1980; Doi and Edwards, 1988; Pavlov et al., 2003; Rubinstein and Colby, 2003; Wehrman et al., 2018). In the dilute regime, polymers do not interact. In the semi-dilute regime, the pervaded volume of the polymers begin to overlap. In the entangled regime, polymer molecules physically interact and entangle (Graessley, 1980; Doi and Edwards, 1988; Pavlov et al., 2003; Rubinstein and Colby, 2003; Wehrman et al., 2018). The overlap concentration, c^*^, is defined in a good solvent as

(1) $$\begin{array}{l} c^{*}=\frac{M}{\frac{4}{3}\pi R_{g}^{3}N_{A}} \end{array}$$

where *M* is the molecular weight, *N*_*A*_ is Avogadro's number and *R*_*g*_ is the radius of gyration defined for a star polymer as $R_{g}^{star}=R_{g}^{arm}{\left[\frac{3f-2}{f}\right]}^{\frac{1}{2}}$ where $R_{g}^{arm}$ is the radius of gyration of a single arm and *f* is the number of functional groups on the polymer. The entanglement concentration, c^**^, is defined as

(2) $$\begin{array}{l} c^{**}=\frac{v\left(M_{s}/N_{A}\right)}{b^{6}} \end{array}$$

where *v* is the excluded volume parameter, *M*_*s*_ is the monomer molecular weight and *b* is the Kuhn length (Graessley, 1980; Doi and Edwards, 1988; Larson, 1999; Pavlov et al., 2003; Rubinstein and Colby, 2003). The gel characterized in this work is a four-arm star PEG-acrylate cross-linked with a linear PEG-dithiol. This is a photopolymerization reaction that uses lithium phenyl-2,4,6-trimethylbenzoylphosphinate (LAP) as a photoinitiator. PEG is chosen due to the versatility of this molecule. PEG is a hydrophilic molecule that can be functionalized with many different chemistries (Bhat and Timasheff, 1992; Iza et al., 1998; Kienberger et al., 2000; Bryant and Anseth, 2002; Hansen et al., 2003; Rubinstein and Colby, 2003; Lee et al., 2007; Aimetti et al., 2009; Fairbanks et al., 2009a). Due to this versatility, PEG has been widely used as the basis for polymeric gel scaffolds that are used in applications from consumer care products to implantable biomaterials. With the development of each new chemistry, the change in the rheology and structure must be understood to design these materials for each application.

Bulk rheological measurements have been the classic way to characterize gelling materials and polymeric solutions (Muthukumar and Winter, 1986; Winter and Chambon, 1986; Chambon and Winter, 1987; Winter, 1987; Adolf and Martin, 1990; Scanlan and Winter, 1991; Izuka et al., 1992; Winter and Mours, 1997; Larson, 1999; Rubinstein and Colby, 2003; Larsen, 2008; Larsen and Furst, 2008; Larsen et al., 2008). In these measurements the viscous, *G*″, and elastic component, *G*′, of the complex modulus, G^*^, is measured. When a material gels the viscous component decreases as the elastic component simultaneously increases. At the gel point, *G*′ and *G*″ are parallel over all frequencies (Muthukumar and Winter, 1986; Winter and Chambon, 1986; Chambon and Winter, 1987; Winter, 1987; Adolf and Martin, 1990; Scanlan and Winter, 1991; Winter and Mours, 1997; Larson, 1999). To determine the point at which a material gels, which is defined as the first sample-spanning network cluster, time-cure superposition (TCS) is used. Time-cure superposition is the superposition of viscoelastic functions at different extents of reaction (Muthukumar and Winter, 1986; Winter and Chambon, 1986; Chambon and Winter, 1987; Winter, 1987; Adolf and Martin, 1990; Scanlan and Winter, 1991; Winter and Mours, 1997; Larson, 1999). TCS is used to determine the critical gel time, *t*_*c*_, and critical relaxation exponent, *n*. The critical relaxation exponent indicates the structure of the scaffold and is a measure similar to a complex modulus, indicating how much energy the scaffold can store and dissipate. Although bulk rheology has been the classic way to collect data of gelling systems, microrheological characterization can also be used.

Microrheological characterization has been used extensively to characterize gelling materials (Freundlich and Seifriz, 1922; Heilbronn, 1922; Seifriz, 1924; Valentine et al., 1996, 2001; Crocker et al., 2000; Gardel et al., 2005; Panorchan et al., 2006; Slopek et al., 2006; Veerman et al., 2006; Caggioni et al., 2007; Wong Po Foo et al., 2009; Corrigan and Donald, 2009a; Mulyasasmita et al., 2011; Schultz and Furst, 2012; Schultz and Anseth, 2013; Furst and Squires, 2017). Our work focuses on passive microrheological characterization of gelling scaffolds. Multiple particle tracking microrheology (MPT), a passive microrheological technique, measures the Brownian motion of probe particles embedded in a material which is related to rheological properties using the Generalized Stokes-Einstein Relation (GSER) (Mason and Weitz, 1995; Crocker and Grier, 1996; Mason, 2000; Valentine et al., 2004; Savin and Doyle, 2005; Squires and Mason, 2010; Furst and Squires, 2017). Advantages of MPT that make it ideal for characterization of gelling scaffolds are: small sample size (1−50 μL), measurement of a large frequency range (up to MHz), short acquisition time enabling measurement of steady state properties of an evolving material and sensitivity in the low moduli range enabling characterization of the fragile microstructure of a gel at the sol-gel transition (Mason et al., 1997; Waigh, 2005, 2016; Squires and Mason, 2010; Schultz and Furst, 2012; Wehrman et al., 2016; Furst and Squires, 2017; Daviran et al., 2018). Additionally, TCS has been adapted to determine the critical gelation time and critical relaxation exponent using MPT measurements (Larsen and Furst, 2008; Larsen et al., 2008; Corrigan and Donald, 2009a,b; Schultz et al., 2009a). Due to these advantages, we use MPT to characterize the critical transitions of PEG-acrylate gels as polymeric interactions are increased.

Previous work determined the overlap concentration, c^*^ = 13 ± 4 wt%, of the PEG-acrylate backbone using bulk rheology (Wehrman et al., 2018). This previous investigation also characterized the change in material properties and structure when the PEG-acrylate backbone is below (3 wt%) and above (10 and 18 wt%) the overlap concentration. This work characterized a change in structure to a more tightly cross-linked scaffold as PEG-acrylate concentration is increased (Wehrman et al., 2018). The present work expands upon this work to determine if there is a gradual or step change in the structure of the material below and above c^*^. Scaffolds are characterized changing the PEG-acrylate concentration below c^*^ from 4 − 9 wt% and above c^*^ at 13 and 15 wt%. Data from the previous study is also included for comparison. First, the kinetics of gelation are measured with MPT. The logarithmic slope of the mean-squared displacement, α, quantifies the change in material properties during scaffold gelation. For this chain-growth gel, there is an initial step when polymers are adding to growing chains and there is no measurable change in the rheology. Once chains begin to cross-link and form the gel network, rapid gelation is measured. Normalization of the UV exposure time with the final time of gelation, defined as the time when α ≈ 0.02, results in all curves collapsing onto a master curve indicating that polymeric interactions do not change the kinetics of gelation. TCS is used to analyze MPT data at PEG-acrylate backbone concentrations above and below c^*^. We calculate a critical relaxation exponent, *n*, and critical gelation time, *t*_*c*_, for each scaffold. The critical relaxation exponent is constant above and below c^*^ with a step change at c^*^. The normalized critical gelation time has no change as a function of PEG-acrylate concentration. Together, these results characterize a change in scaffold structure that is only dependent on the overlap concentration. This will enable the scaffold rheological properties to be tailored without changing the structure. This work provides additional information about scaffold properties and structure which can be used to tailor these materials for applications that include decreasing permeability during enhanced oil recovery, rheological modification of fabric and home care products and as synthetic implantable materials for wound healing.

## Materials and Methods

### Hydrogel Scaffold

The hydrogel scaffold is composed of a four-arm star PEG-acrylate which is cross-linked with PEG-dithiol. PEG-acrylate has a molecular weight of *M*_*n*_ = 20, 000 g mol^−1^ (JenKem Technology) and is end-functionalized with acrylates (*f* = 4 where *f* is functionality). The cross-linker is a PEG end-functionalized with thiols (*M*_*n*_ = 1, 500 g mol^−1^, *f* = 2, Aldrich). These two molecules undergo a chain-growth polymerization upon exposure to ultraviolet (UV) light (PhotoFluor LM-75, output range 340 − 800 nm, 89 North, Inc.). The reaction is initiated by lithium phenyl-2,4,6 trimethylbenzoylphosphinate (LAP). LAP is synthesized using previously published protocols (Fairbanks et al., 2009a). Carboxylated polystyrene probe particles (2*a* = 0.97 ± 0.01 μm where *a* is the particle radius, Polysciences, Inc.) are added to the polymer precursor solution to enable multiple particle tracking microrheological measurements.

Hydrogel samples maintain an acrylate:thiol ratio of 1.4:1. The concentration of PEG-acrylate is changed by 1 wt% from 4−9 wt%. Higher PEG-acrylate concentrated samples are measured and are 13 and 15 wt%. Data from 3, 10 and 18 wt% are included from a previous study for comparison (Wehrman et al., 2018). During sample preparation, all concentrated stock solutions are kept on ice for 30 min prior to addition to the polymer precursor solution. This ensures that there are no reactions in the solutions, especially thiol-thiol reactions in the PEG-dithiol stock solution. The polymer precursor solutions consist of PEG-acrylate, PEG-dithiol, 1.5 mM LAP, 0.052% solids per volume probe particles and water. Solutions are well-mixed prior to injection into the sample chamber. Hydrogel samples are gelled by exposure to UV light and MPT data are collected.

### Multiple Particle Tracking Microrheology

Multiple particle tracking microrheology (MPT) measures the change in rheological properties during scaffold gelation. In MPT, the Brownian motion of 1 μm fluorescently labeled probe particles (carboxylated polystyrene particles, 2*a* = 0.97 ± 0.01 μm, Polysciences, Inc.) is measured. Data are collected using video microscopy on an inverted fluorescent microscope (Zeiss Observer Z1, Carl Zeiss AG). A high numerical aperture water-immersion objective is used to capture data, which maximizes the pixels per probe particle (63 × water-immersion objective, N.A. 1.3, 1 × optovar, Carl Zeiss AG). Our equipment is calibrated to minimize static and dynamic particle tracking errors (Savin and Doyle, 2005). Data are collected at 30 frames per second and an exposure time of 1000 μs (Phantom Miro M120, 1024 × 1024 pixels, Vision Research Inc.).

After data acquisition, the brightness-weighted centroid of each probe particle is determined using classical tracking algorithms (Crocker and Grier, 1996; Crocker and Weeks, 2011; Furst and Squires, 2017). These algorithms determine the center of each particle in each frame. Those particle centers are linked together into trajectories using a probability distribution function that accounts for Brownian motion (Crocker and Grier, 1996; Crocker and Weeks, 2011; Furst and Squires, 2017). From the particle positions, the ensemble-averaged mean-squared displacement (MSD, 〈Δ*r*^2^(τ)〉) is calculated from the two-dimensional data using 〈Δ*r*^2^(τ)〉 = 〈Δ*x*^2^(τ)〉 + 〈Δ*y*^2^(τ)〉. From the MSD, rheological properties can be calculated using the Generalized Stokes-Einstein Relation

(3) $$\begin{array}{l} \langle \Delta r^{2}\left(\tau \right)\rangle =\frac{k_{B}T}{\pi a}J\left(\tau \right) \end{array}$$

where *k*_*B*_ is the Boltzmann constant, *T* is the temperature, *a* is the particle radius and *J*(τ) is the creep compliance.

To measure gelation using MPT we first add probe particles into the polymer precursor solution. Briefly, 1 μm probe particles are washed 3 × by dilution and centrifugation at 5, 000 RPM for 5 min (Centrifuge 5425, Eppendrof) to remove any excess dye. Probe particles are then sonicated (40 kHz, Emerson Industrial Automation) for 15 min to ensure that there are no particle aggregates. We add probes at a final concentration of 0.052% solids per volume to the polymer precursor solution. We then inject our polymer precursor solution into a sample chamber. Sample chambers are made on a standard glass slide (75 × 25 × 1 mm, Thermo Fisher Scientific), with 0.16 mm thick spacers and a coverglass (22 × 22 × 0.16 mm) as the top of the chamber. After the polymer precursor solution has filled the sample chamber it is sealed with an air-curing epoxy (Gorilla Glue Company). During curing, sample chambers are kept in the dark and allowed to cure for 15 min.

Each sample is exposed to UV light and MPT data are collected. This is repeated until ≈30 min after complete gelation is measured. Neutral density filters (Chroma Technology) are used to lower the intensity of the UV light. This is done to slow the gelation reaction enabling acquisition of MPT data during the sol-gel transition. The neutral density filters used are 32 and 10% transmission (Chroma Technologies) for 4−15 and 18 wt% PEG-acrylate, respectively. Due to the arbitrary time of UV exposure, data are reported as a normalized UV exposure time which is UV exposure time divided by final time of gelation. The final time of gelation is the time when the logarithmic slope of the MSD, α, is ≤0.02. All MPT experiments are repeated at least 3× to ensure repeatability and critical values are reported as the average ± the standard deviation.

## Results and Discussion

This work characterizes the change in rheological properties and scaffold structure as polymeric interactions are increased. Previous work determined the overlap concentration, c^*^, of this PEG-acrylate backbone using bulk rheology and MPT. The value of c^*^ = 13 ± 4 wt% (Wehrman et al., 2018). We consider scaffolds with PEG-acrylate concentrations *c* ≤ 9 wt% to be below c^*^ and *c* > 9 wt% to be above c^*^. This uses the lower limit of c^*^ as the transition point. This value is chosen not only from the measurements of c^*^ but also from the characterization of scaffold properties presented here. We measure the same trend in gelation kinetics as a function of UV exposure, regardless of PEG-acrylate concentration. Previous work found that below the overlap concentration the material had a more open structure than samples at and above the overlap concentration. This work expands this initial study determining that there is a step change in the scaffold structure when the overlap concentration is reached. In the previous study, we hypothesized that there would be a gradual decrease in the value of *n* below the overlap concentration and a constant value above c^*^. The gradual decrease in *n* below c^*^ would have indicated a more densely cross-linked structure as the concentration is increased. Instead, we measure no change in the dilute, *c* < c^*^, and semi-dilute, *c* > c^*^, concentration regimes, but there is a step change in the structure of the scaffold at the transition. This result indicates that the structure remains constant above and below the overlap concentration regardless of PEG-acrylate concentration. This information can lead to the design of new materials, where the moduli of the material can be tailored for applications, such as the mimic of a tissue or a material to reduce permeability in enhanced oil recovery without changing the scaffold structure.

### Scaffold Gelation

Multiple particle tracking microrheology measures the scaffold properties during gelation. After UV exposure, MPT data are collected. From the MPT data, the ensemble-averaged mean-squared displacement is calculated for all the measured probe particles. Previous work that used MPT to measure this hydrogel scaffold determined that the scaffold evolves homogeneous during gelation with each particle probing the same material properties (Wehrman et al., 2018). The logarithmic slope of the MSD, $\alpha =\frac{d\log\langle \Delta r^{2}\left(\tau \right)\rangle }{d\log\tau }$, is a measure of the state of the material (Stauffer et al., 1982; Winter and Chambon, 1986; Adolf and Martin, 1990; Larsen and Furst, 2008; Corrigan and Donald, 2009a,b; Schultz et al., 2009a, 2012; Schultz and Furst, 2012; Wehrman et al., 2016; Daviran et al., 2018). When α = 1 probe particles are freely diffusing and the material is a liquid. When α → 0 probe particles are completely arrested in the gel scaffold. This occurs at a low moduli value, *G*′ ≈ 4 Pa (Waigh, 2005; Schultz and Furst, 2011, 2012; Furst and Squires, 2017). Due to the low maximum moduli value, bulk rheology should be used to supplement MPT to measure the equilibrated material properties. Finally, when 0 < α < 1 the material is a viscoelastic sol or gel and probe particle movement is restricted.

To quantitatively determine when the sol-gel transition occurs, time-cure superposition (TCS) is used to analyze the critical transition and will be discussed in detail below. From TCS, the critical relaxation exponent is determined, *n*. α = *n* is the sol-gel transition (Larsen and Furst, 2008; Corrigan and Donald, 2009a,b; Schultz et al., 2009a,b; Schultz and Anseth, 2013; Adibnia and Hill, 2016; Wehrman et al., 2016; Escobar et al., 2017; Daviran et al., 2018). This is when the first sample-spanning network cluster has formed and is the definition of a gel (Stauffer et al., 1982; Muthukumar and Winter, 1986; Winter and Chambon, 1986; Chambon and Winter, 1987; Winter, 1987; Adolf and Martin, 1990; Larsen and Furst, 2008; Corrigan and Donald, 2009a,b; Schultz et al., 2009a,b; Schultz and Anseth, 2013; Adibnia and Hill, 2016; Wehrman et al., 2016; Escobar et al., 2017; Daviran et al., 2018). To determine the state of the material α is compared to *n*. When α < *n* the material is a gel and when α > *n* the material is a sol (Larsen and Furst, 2008; Corrigan and Donald, 2009a,b; Schultz et al., 2009a,b; Schultz and Anseth, 2013; Adibnia and Hill, 2016; Wehrman et al., 2016; Escobar et al., 2017); (Daviran et al., 2018).

**Figure 1:**
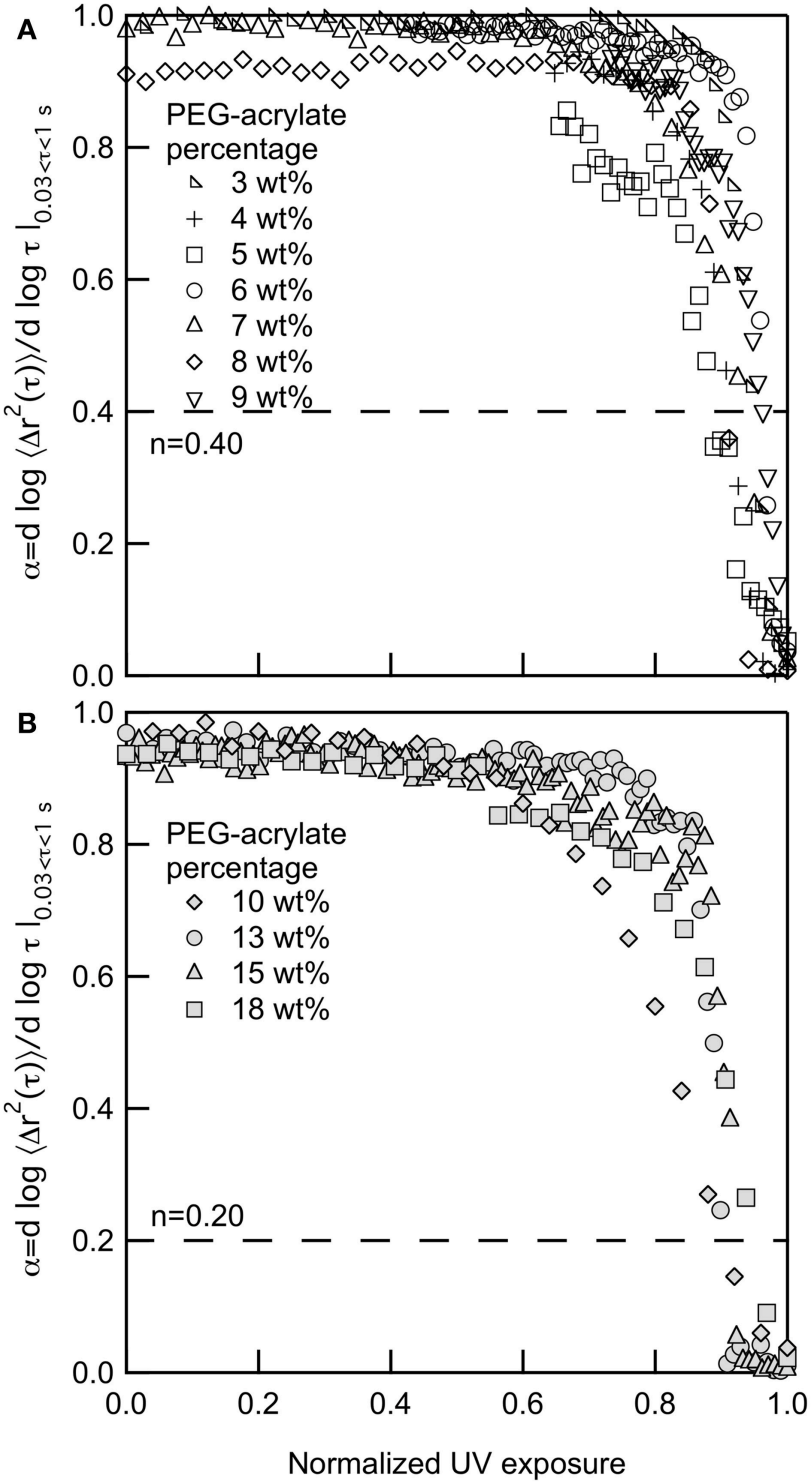
Logarithmic slope of the MSD, α, for gelation of PEG-acrylate gels when the backbone concentrations is **(A)** below and **(B)** above the overlap concentration, c^*^. The dotted line in both graphs is the critical relaxation exponent, *n*. 3, 10 and 18 PEG-acrylate wt% data originally appeared in Wehrman et al. (2018).

Figures 1A,B show the change in α as the polymer precursor solution is exposed to UV light. Upon initial exposure to UV light there is lag prior to scaffold gelation. This lag is due to radicals being formed in the polymer precursor solution and the growth of polymeric chains that are not cross-linking into a network structure. During chain-growth, cross-linking occurs by the addition of polymers to growing network chains. Those chains then cross-link together to form the scaffold network structure (Rubinstein and Colby, 2003; Tibbitt et al., 2013; Payamyar et al., 2016). This cross-linking of chains leads to the steep slope in α vs. normalized UV exposure. Prior to the decrease in α, probe particles are freely diffusing. During this time polymer chains are forming but are not large enough to restrict probe particle motion. When these chains start to cross-link, the network structure is forming and probe particle movement becomes restricted and, eventually, arrested in the gel scaffold. At the point of probe particle arrest, the network structure will continue to grow, but MPT can no longer measure the change in material properties.

For all PEG-acrylate samples the kinetics of gelation follow the same trend that is described above. Figure 1A shows PEG-acrylate samples with concentrations below c^*^ and Figure 1B are concentrations above c^*^. This is further illustrated in Figure S1, where all concentrations are plotted together. In Figures 1A,B, UV exposure time is normalized by the UV exposure when complete gelation is measured, defined as α ≤ 0.02. This value is used because it is a slope where no probe particle movement is measured. Upon normalization, all data sets collapse and have a lag in gelation and then a steep slope, where the value of α decreases rapidly with time. Therefore, from these experiments, we can conclude that there is no change in the mechanism of gelation when polymeric interactions are added into the system by concentrating the PEG-acrylate backbone over c^*^.

### Time-Cure Superposition

Time-cure superposition (TCS) is used to analyze each scaffold gelation reaction and determine the critical relaxation exponent, *n*, and the critical gelation time, *t*_*c*_. TCS is the superposition of viscoelastic functions at different extents of reaction (Muthukumar and Winter, 1986; Winter and Chambon, 1986; Chambon and Winter, 1987; Winter, 1987; Adolf and Martin, 1990; Larsen and Furst, 2008; Corrigan and Donald, 2009a,b; Schultz et al., 2009a,b; Schultz and Anseth, 2013; Adibnia and Hill, 2016; Wehrman et al., 2016; Escobar et al., 2017; Daviran et al., 2018). This analysis exploits the self-similarity of measurements of the scaffold material properties prior to and after gelation to shift data onto master curves. In MPT, the shortest lag times, 0.03 ≤ τ ≤ 1s, measure the longest relaxation times, τ_*R*_, of the material. This is because the short lag times are equivalent to high frequency measurements in bulk rheology. The longest relaxation times in the pre- and post-gel give distinct curvature to MSDs measured with MPT. In the pre-gel, the longest relaxation time of the polymers are measured. In the post-gel, the longest relaxation time of the gel network is being probed (Muthukumar and Winter, 1986; Winter and Chambon, 1986; Chambon and Winter, 1987; Winter, 1987; Adolf and Martin, 1990; Larsen and Furst, 2008; Corrigan and Donald, 2009a,b; Schultz et al., 2009a,b; Schultz and Anseth, 2013; Adibnia and Hill, 2016; Wehrman et al., 2016; Escobar et al., 2017; Daviran et al., 2018). Since these measurements are probing the same relaxation time, over a large time scale, the data can be shifted into pre- and post-gel master curves by shifting along the lag time and MSD axes.

The shift factors determine the critical values, namely the critical relaxation exponent and critical gelation time (Muthukumar and Winter, 1986; Winter and Chambon, 1986; Chambon and Winter, 1987; Winter, 1987; Adolf and Martin, 1990; Larsen and Furst, 2008; Corrigan and Donald, 2009a,b; Schultz et al., 2009a,b; Schultz and Anseth, 2013; Adibnia and Hill, 2016; Wehrman et al., 2016; Escobar et al., 2017; Daviran et al., 2018). The lag time shift factor, *a*, is related to the inverse of the longest relaxation time, τ_*R*_, and the distance away from the critical gelation time, $\frac{\left|t-t_{c}\right|}{t_{c}}$, by a scaling factor *y* by

(4) $$\begin{array}{l} a\sim \tau_{R}^{-1}\sim {\left(\frac{\left|t-t_{c}\right|}{t_{c}}\right)}^{y}. \end{array}$$

Similarly the MSD shift factor, *b*, is related to the inverse of the steady state creep compliance, *J*_*e*_, and the distance away from the critical gelation time by a scaling factor *z* by

(5) $$\begin{array}{l} b\sim J_{e}^{-1}\sim {\left(\frac{\left|t-t_{c}\right|}{t_{c}}\right)}^{z}. \end{array}$$

The critical relaxation exponent is the ratio of the two scaling exponent

(6) $$\begin{array}{l} n=\frac{z}{y}. \end{array}$$

The critical relaxation exponent determines the state of the material as described in detail above. This is the value of α where the first sample-spanning network cluster forms during gelation. Additionally, *n* can be thought of as a complex modulus, G^*^, with both a viscous and elastic component. When *n* > 0.5 the material is an open porous network which dissipates more energy than it stores. When *n* < 0.5 the scaffold is a tightly cross-linked network that stores more energy. When *n* = 0.5 the scaffold is a percolated network which stores and dissipates equal amounts of energy (Stauffer et al., 1982; Schultz et al., 2009a; Wehrman et al., 2018).

**Figure 2:**
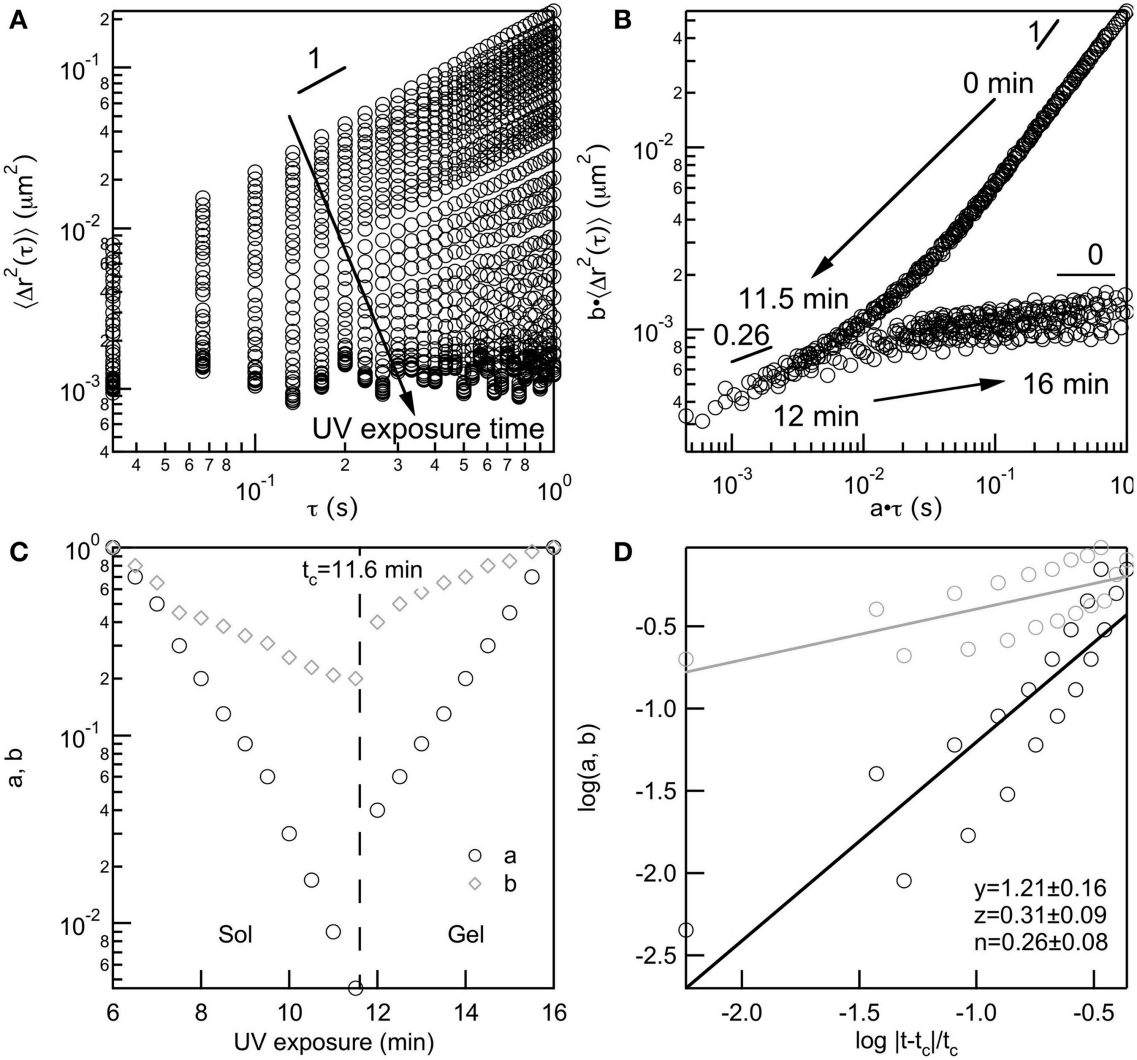
Four wt% PEG-acrylate scaffold gelation analyzed using time-cure superposition. **(A)** Ensemble-averaged MSD data collected as UV exposure time is increased. **(B)** MSD curves shifted into pre- and post-gel master curves using shifting factors *a* and *b*, which **(C)** diverge at the critical gelation time, *t*_*c*_, at the sol-gel transition. **(D)** The scaling exponents *y* and *z* are determined from the slope of log *a* and log *b* vs. the logarithm of the distance away from the critical gelation time, $\log\frac{\left|t-t_{c}\right|}{t_{c}}$. The critical relaxation exponent, *n* is calculated from the scaling factors.

All data taken of PEG-acrylate gelation is analyzed with TCS to determine the value of the critical relaxation exponent, *n*, and the critical gelation time, *t*_*c*_. TCS for a 4 wt% PEG-acrylate gelation is shown in Figure 2. An example of TCS for all other PEG-acrylate concentrations are in the Supplementary Material, Figures S2–S8. The ensemble-averaged MSD is calculated from MPT data, Figure 2A. The MSDs decrease in both magnitude and α as UV exposure time is increased. Initially, probe particles are freely diffusing in the polymer solution. As UV exposure increases the scaffold network grows and probe particle movement decreases. As mentioned above, at the sol-gel transition there is a change in the shape of MSD curves due to the change in the relaxation times measured from the relaxation of polymers to the relaxation of a network. This change in shape enables the MSD curves to be shifted into two master curves, a pre- and post-gel master curve, Figure 2B. The start of the pre-gel master curve is the polymeric solution with α = 1. As the value of *a*·τ decreases the value of α also decreases which is due to the growing polymer chains restricting the movement of the probe particles. Additionally, the curvature of the pre-gel curve shows that at short lag times the MSD curves up slightly, which is the measure of the polymeric longest relaxation time, τ_*R*_ (Furst and Squires, 2017; Wehrman et al., 2018). At α = *n* the pre- and post-gel master curves meet. In the pre-gel curve this is the last measurement before the sample-spanning network cluster forms and in the post-gel curve this is the first measurement after the sample-spanning network cluster has formed. In the post-gel master curve as *a*·τ increases, there is a decrease in α until α → 0 and probe particles are completely arrested in the polymerized network scaffold. In the post-gel master curve, the curvature is reversed and at short lag times, the MSDs curve down, indicative of measurements of the longest relaxation time of a network.

The shift factors are used to determine the critical relaxation exponent and critical gelation time. In Figure 2C both the lag time and MSD shift factors diverge at the sol-gel transition. This is the divergence of the viscosity upon network formation and the emergence of the elastic modulus, *G*′, as the network continues to grow. This divergence happens at the critical gelation time, which is *t*_*c*_ = 11.6 min for this 4 wt% PEG-acrylate network. The critical relaxation exponent is the ratio of scaling exponents. Scaling exponents *y* and *z* are calculated by fitting the log *a* and log *b* vs. the $\log\frac{\left|t-t_{c}\right|}{t_{c}}$, Figure 2D. The ratio of the scaling exponents determine the critical relaxation exponent, which is *n* = 0.26 ± 0.08 for this hydrogel scaffold.

This analysis is done for all MSD data collected both above and below c^*^. Since the absolute value of UV exposure can be dependent on the size of the sample, we normalize the critical gelation time by the final time of gelation $\left(t_{c,norm}=\frac{t_{c}}{t_{final}}\right)$. The critical gelation time, *t*_*c*_, is defined as the time when the first sample-spanning network cluster is formed in the material. The final time of gelation, *t*_*final*_, is again defined as the time when α ≤ 0.02. The normalized critical gelation time is *t*_*c, norm*_ = 0.86 ± 0.17 and *t*_*c, norm*_ = 0.96 ± 0.06 for PEG-acrylate concentrations below and above c^*^, respectively. A plot of these values is provided in the Supplementary Material, Figure S9. These values are within error of each other, indicating that the critical gelation time occurs at the same point within the scaffold gelation reaction regardless of the backbone concentration. The first sample-spanning cluster will require a similar amount of cross-links regardless of polymeric interactions and, therefore, occurs at a similar point in the gelation process. This is also supported by the collapse of MPT measurements throughout gelation in Figures 1A,B. These results show that scaffold gelation proceeds through the same reaction mechanism regardless of polymeric interactions in the precursor solution.

**Figure 3:**
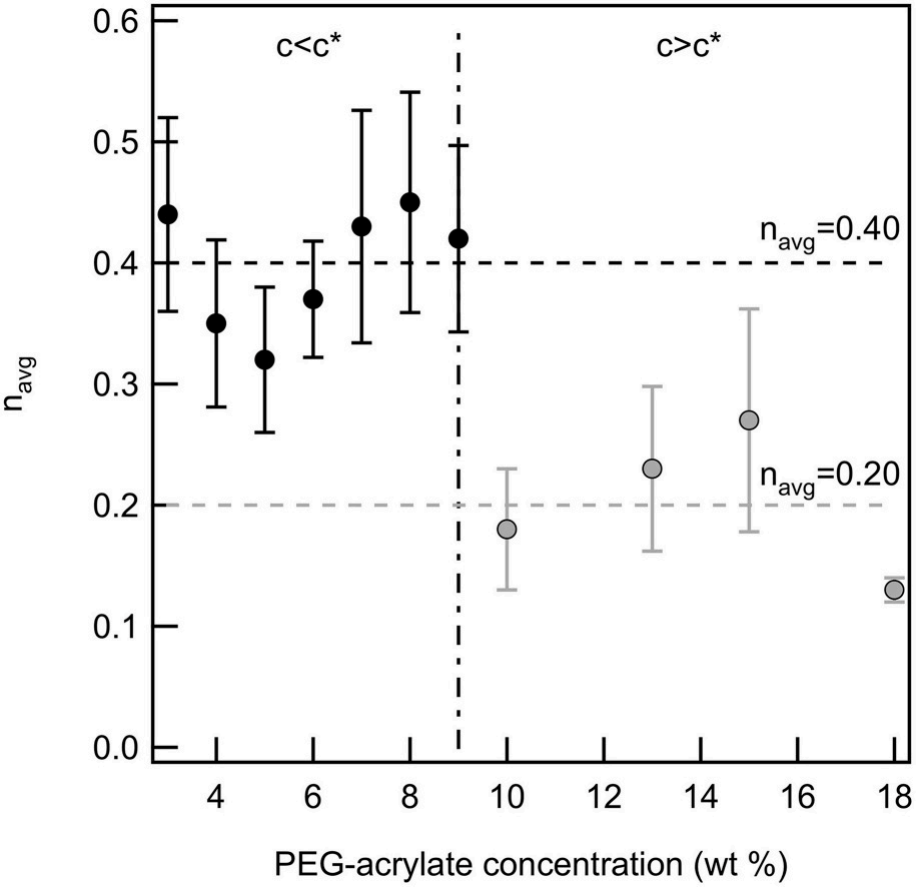
The critical relaxation exponent below and above c^*^ for a PEG-acrylate:PEG-dithiol scaffold by increasing the backbone concentration above c^*^. There is a step change in the value of *n* when the concentration is above c^*^.

Finally, the critical relaxation exponent is calculated for scaffolds above and below the overlap concentration of PEG-acrylate. Figure 3 is a plot of the average value of *n* vs. PEG-acrylate concentration. As described previously, scaffold with *c* ≤ 9 wt% are considered below c^*^ and *c* > 9 wt% are considered above c^*^. The critical relaxation exponent indicates the scaffold structure and is a measure similar to a complex modulus, indicating how much energy the scaffold can store and dissipate. The value of *n* has a step change at the overlap concentration. Below c^*^ *n*_*avg*_ = 0.40 ± 0.03 and above c^*^ *n*_*avg*_ = 0.20 ± 0.03. This step change in the value of *n* indicates that there is a change in structure below and above the overlap concentration, but within the dilute and semi-dilute concentration regimes there is no structural change. For all concentrations, a tightly cross-linked network that stores more energy than it dissipates is measured. When *c* > c^*^ the network is more tightly cross-linked with a smaller pore structure than when *c* < c^*^. This is due to polymeric interactions. When *c* > c^*^, polymers are interacting and are able to enter into the pervaded volume of other chains. Due to the polymers being physically closer during network formation there is a high likelihood of arm interpenetration during network formation. This leads to a scaffold with a smaller porous structure that can store energy. This result indicates that the scaffold structure is unchanged by the change in backbone concentration. The only factor that changes scaffold structure is whether the backbone concentration is above or below c^*^. Therefore, this scaffold can be tailored to change the elastic moduli of the material without changing the structure of the scaffold enabling the material to be tailored for desired applications.

## Conclusions

This work characterizes the change in rheological properties and scaffold structure during the photopolymerization of a four-arm star PEG-acrylate:PEG-dithiol hydrogel scaffold as polymeric interactions are added to the system. MPT is used to measure the change in scaffold properties as UV exposure time is increased. The gelation mechanism is the same for all scaffolds, regardless of polymeric interactions. The rheology of all scaffolds indicate that they follow a typical chain-growth polymerization. Upon UV exposure, chains of polymers begin to grow in the system and gelation is measured when these chains begin to cross-link into a network. Measured mean-squared displacements are further analyzed using time-cure superposition. The critical relaxation exponent and critical gelation time are determined. The normalized critical gelation time is independent of polymeric interactions. This result supports measurements that all scaffolds follow the same gelation mechanism. The critical relaxation exponent is sensitive to polymeric interactions. A step change in the value of the critical relaxation exponent is measured when polymeric interactions increase above the overlap concentration. This decrease in the value of *n* indicates that scaffolds made with backbone concentrations above c^*^ have a smaller porous network which stores more energy than scaffold made with PEG-acrylate concentrations below c^*^. This is due to the increased physical interactions, which likely lead to an interpenetrated network structure when *c* > c^*^.

The results of this work provide a predictability of material properties, structure and kinetics during gelation. This information can be used to determine the feasibility of these scaffolds for desired applications, particularly when these materials are made in a new environment and require a specific modulus and structure. A stable structure below and above c^*^ enables properties of the material to be tailored while the structure remains constant. The wide applicability of hydrogel scaffolds has lead to the need to more precisely design these materials. This work informs this design by building a knowledge base which can be exploited to minimize trial-and-error when a scaffold is used in a new application.

## Author Contributions

HZ was responsible for design of experiments and collection and analysis of data. MW was responsible for design of experiments. KS was responsible for design of experiments, data analysis and writing the manuscript.

### Conflict of Interest Statement

The authors declare that the research was conducted in the absence of any commercial or financial relationships that could be construed as a potential conflict of interest.

## Supplementary Material

Additional graphs for α vs. normalized UV exposure for all PEG-acrylate concentrations, TCS of each PEG-acrylate concentration and *t*_*c, norm*_ for all PEG-acrylate concentrations are provided. Additionally, a table with the values of *n* and *t*_*c, norm*_ are provided for each PEG-acrylate concentration and can be found in the Supplemental data.
