# Supplementary material for: Structural Changes in Polymeric Gel Scaffolds Around the Overlap Concentration
Source: Front Chem. 2019 May 8;7:317. doi: 10.3389/fchem.2019.00317 (PMC6517517; doi:10.3389/fchem.2019.00317)
Supplement: Supplementary file 1 [file Data_Sheet_1.pdf]

Supplementary Material: Structural Changes in Polymeric Gel Scaffolds Around the Overlap Concentration

Han Zhang [1], Matthew D. Wehrman [1] and Kelly M. Schultz [1,*]

[1]Department of Chemical and Biomolecular Engineering, Lehigh University, Bethlehem, PA, USA

* Corresponding author: 111 Research Dr., Iacocca Hall, Bethlehem, PA 18015, USA, Email: kes513@lehigh.edu

SUPPLEMENTARY TABLES AND FIGURES

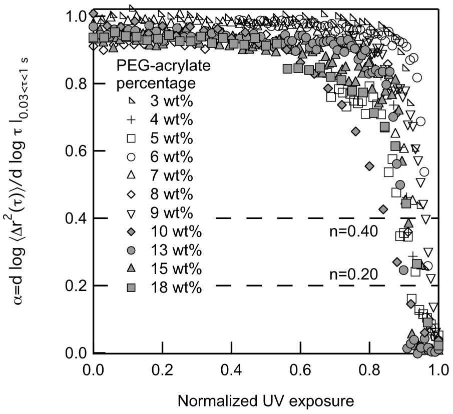

Figure S1. Logarithmic slope of the MSD, α, measured during gelation of PEG-acrylate gels when the backbone concentrations is below and above the overlap concentration, c^*. 3, 10 and 18 PEG-acrylate wt$\%$ data originally appeared in Wehrman et al., *AIChE Journal*, **64** (8), 3168−3176, 2018.

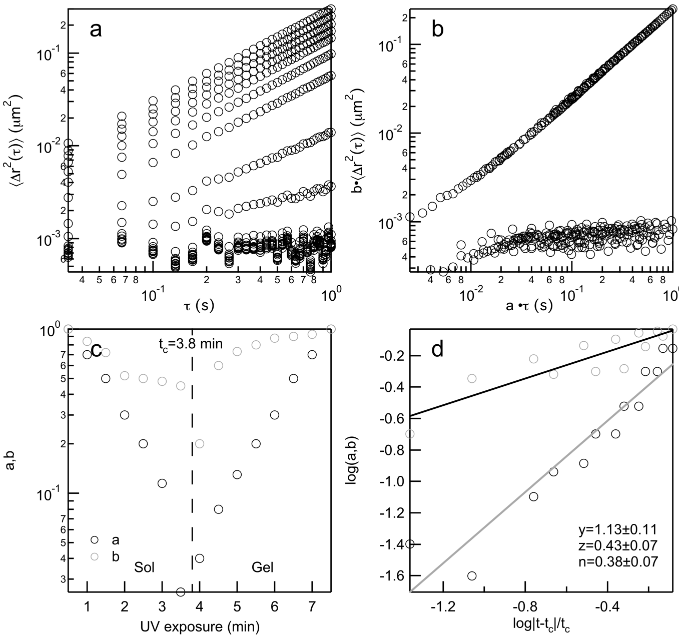

Figure S2. 5 wt% PEG-acrylate scaffold (below c^*) gelation analyzed using time-cure superposition. (a) Ensemble-averaged MSD data that is shifted into (b) pre- and post-gel master curves using shifting factors a and b. (c) These shift factors diverge at the critical gelation time, t_c, at the sol-gel transition. (d) The scaling exponents y and z are determined from the slope of $\log a$ and $\log b$ versus the logarithm of the distance away from the critical gelation time, $\log \frac{|t-t_c|}{t_c}$. The critical relaxation exponent, n is calculated from the scaling factors.

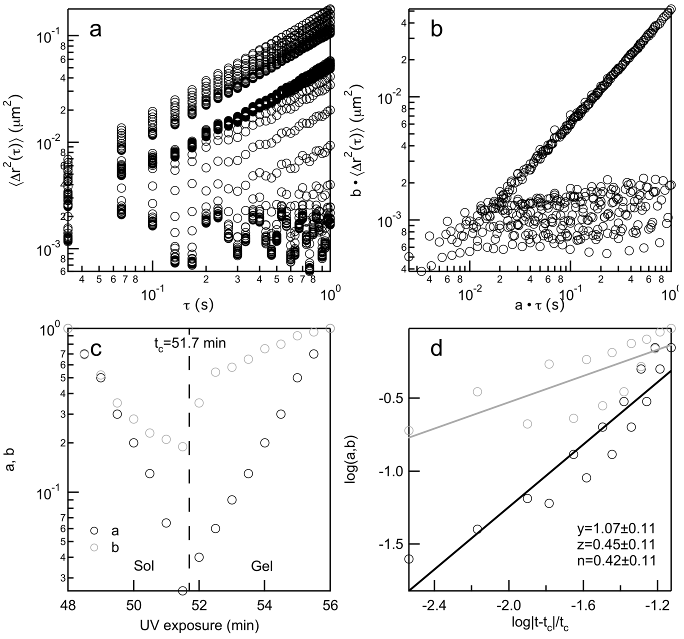

Figure S3. 6 wt% PEG-acrylate scaffold (below c^*) gelation analyzed using time-cure superposition. (a) Ensemble-averaged MSD data that is shifted into (b) pre- and post-gel master curves using shifting factors a and b. (c) These shift factors diverge at the critical gelation time, t_c, at the sol-gel transition. (d) The scaling exponents y and z are determined from the slope of $\log a$ and $\log b$ versus the logarithm of the distance away from the critical gelation time, $\log \frac{|t-t_c|}{t_c}$. The critical relaxation exponent, n is calculated from the scaling factors.

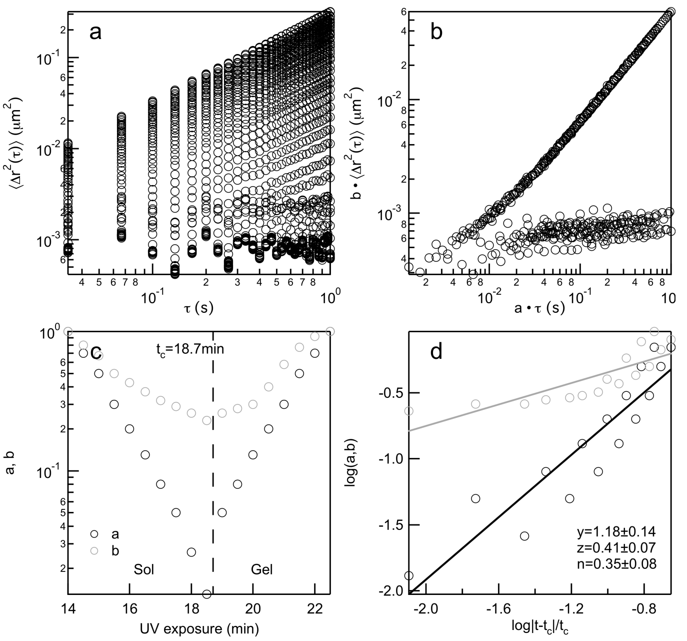

Figure S4. 7 wt% PEG-acrylate scaffold (below c^*) gelation analyzed using time-cure superposition. (a) Ensemble-averaged MSD data that is shifted into (b) pre- and post-gel master curves using shifting factors a and b. (c) These shift factors diverge at the critical gelation time, t_c, at the sol-gel transition. (d) The scaling exponents y and z are determined from the slope of $\log a$ and $\log b$ versus the logarithm of the distance away from the critical gelation time, $\log \frac{|t-t_c|}{t_c}$. The critical relaxation exponent, n is calculated from the scaling factors.

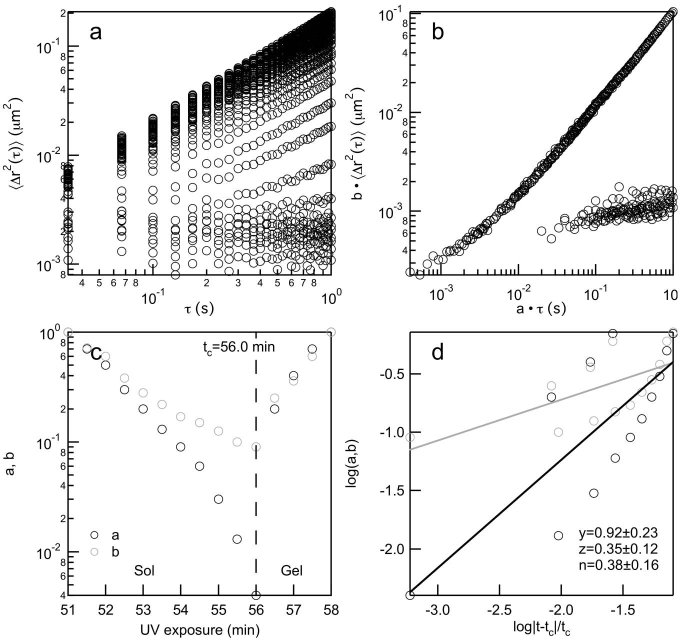

Figure S5. 8 wt% PEG-acrylate scaffold (below c^*) gelation analyzed using time-cure superposition. (a) Ensemble-averaged MSD data that is shifted into (b) pre- and post-gel master curves using shifting factors a and b. (c) These shift factors diverge at the critical gelation time, t_c, at the sol-gel transition. (d) The scaling exponents y and z are determined from the slope of $\log a$ and $\log b$ versus the logarithm of the distance away from the critical gelation time, $\log \frac{|t-t_c|}{t_c}$. The critical relaxation exponent, n is calculated from the scaling factors.

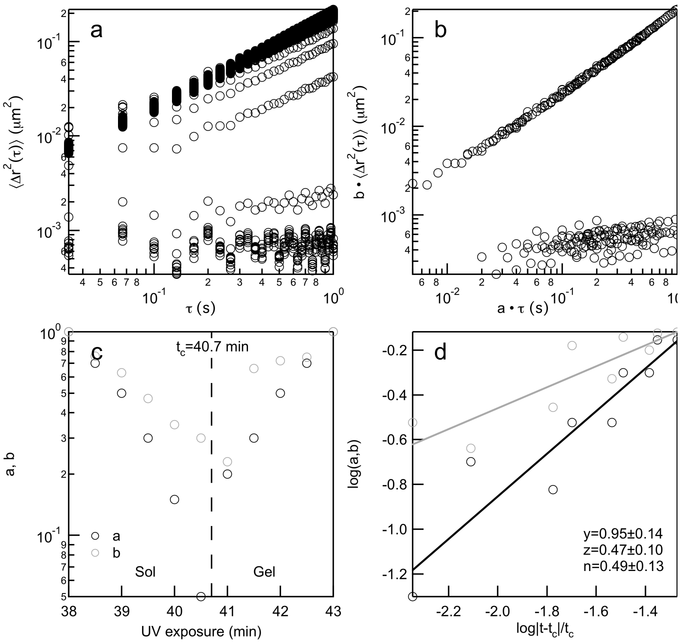

Figure S6. 9 wt% PEG-acrylate scaffold (below c^*) gelation analyzed using time-cure superposition. (a) Ensemble-averaged MSD data that is shifted into (b) pre- and post-gel master curves using shifting factors a and b. (c) These shift factors diverge at the critical gelation time, t_c, at the sol-gel transition. (d) The scaling exponents y and z are determined from the slope of $\log a$ and $\log b$ versus the logarithm of the distance away from the critical gelation time, $\log \frac{|t-t_c|}{t_c}$. The critical relaxation exponent, n is calculated from the scaling factors.

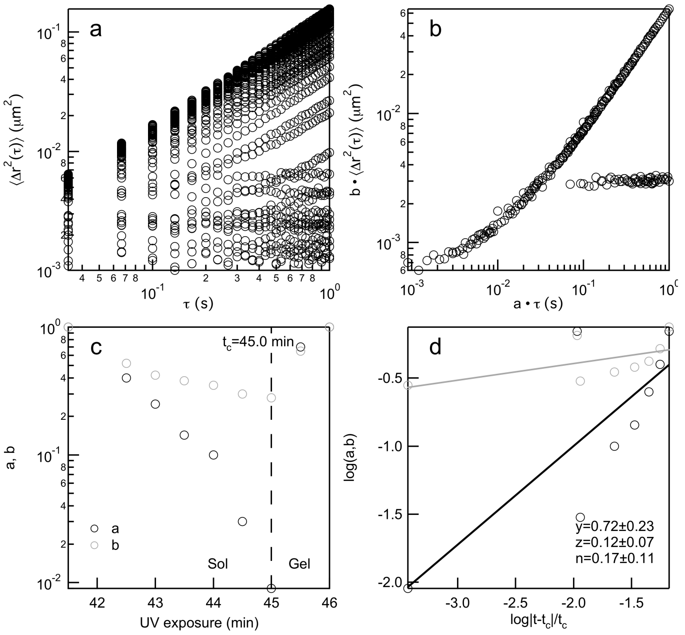

Figure S7. 13 wt% PEG-acrylate scaffold (above c^*) gelation analyzed using time-cure superposition. (a) Ensemble-averaged MSD data that is shifted into (b) pre- and post-gel master curves using shifting factors a and b. (c) These shift factors diverge at the critical gelation time, t_c, at the sol-gel transition. (d) The scaling exponents y and z are determined from the slope of $\log a$ and $\log b$ versus the logarithm of the distance away from the critical gelation time, $\log \frac{|t-t_c|}{t_c}$. The critical relaxation exponent, n is calculated from the scaling factors.

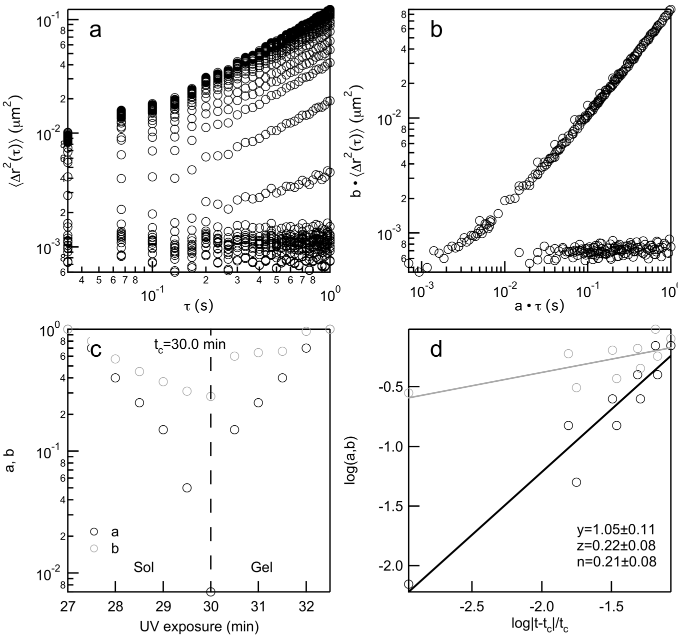

Figure S8. 15 wt% PEG-acrylate scaffold (above c^*) gelation analyzed using time-cure superposition. (a) Ensemble-averaged MSD data that is shifted into (b) pre- and post-gel master curves using shifting factors a and b. (c) These shift factors diverge at the critical gelation time, t_c, at the sol-gel transition. (d) The scaling exponents y and z are determined from the slope of $\log a$ and $\log b$ versus the logarithm of the distance away from the critical gelation time, $\log \frac{|t-t_c|}{t_c}$. The critical relaxation exponent, n is calculated from the scaling factors.

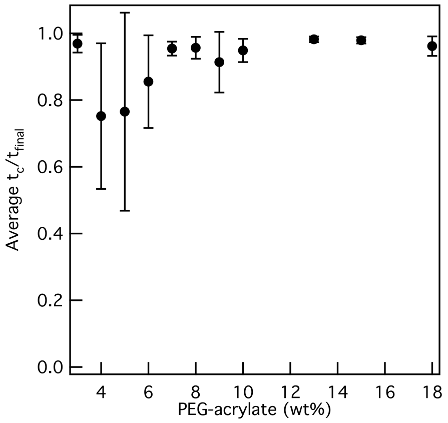

Figure S9. Average $t_{c,norm} = \frac{t_c}{t_{final}}$ for all PEG-acrylate backbone concentrations determined using time-cure superposition. $t_{c,norm}$ is independent of polymeric interactions.

Table S1. Average critical relaxation exponents, n_{avg}, and normalized critical gelation time, $t_{c,norm}$. All numbers are reported as the average value $\pm$ the standard deviation. Data for 3, 10 and 18 wt% data originally appeared in Wehrman et al., *AIChE Journal*, **64** (8), 3168−3176, 2018.

PEG-acrylate wt%	n_{avg}	$t_{c,norm}$
3 wt%	0.44 ± 0.08	0.97 ± 0.03
4 wt%	0.35 ± 0.07	0.75 ± 0.22
5 wt%	0.32 ± 0.06	0.77 ± 0.30
6 wt%	0.37 ± 0.05	0.85 ± 0.14
7 wt%	0.43 ± 0.10	0.95 ± 0.02
8 wt%	0.45 ± 0.09	0.96 ± 0.03
9 wt%	0.42 ± 0.08	0.91 ± 0.09
10 wt%	0.18 ± 0.05	0.95 ± 0.03
13 wt%	0.23 ± 0.07	0.98 ± 0.01
15 wt%	0.27 ± 0.09	0.98 ± 0.01
18 wt%	0.13 ± 0.01	0.96 ± 0.03
